# The Elicitation of Relaxation and Interoceptive Awareness Using Floatation Therapy in Individuals With High Anxiety Sensitivity

**DOI:** 10.1016/j.bpsc.2018.02.005

**Published:** 2018-03-09

**Authors:** Justin S. Feinstein, Sahib S. Khalsa, Hung Yeh, Obada Al Zoubi, Armen C. Arevian, Colleen Wohlrab, Marie K. Pantino, Laci J. Cartmell, W. Kyle Simmons, Murray B. Stein, Martin P. Paulus

**Affiliations:** Laureate Institute for Brain Research (JSF, SSK, HY, OAZ, CW, MKP, WKS, MPP); Oxley College of Health Sciences (JSF, SSK, LJC, WKS), University of Tulsa; Gallogly College of Engineering (OAZ), University of Oklahoma, Tulsa, Oklahoma; Department of Psychiatry and Biobehavioral Sciences (ACA), University of California Los Angeles, Los Angeles; and Department of Psychiatry (MBS), University of California San Diego, La Jolla, California

**Keywords:** Anxiety, Blood pressure, Floatation-REST, Floating, Interoception, Mindfulness, Novel intervention, Relaxation response

## Abstract

**BACKGROUND:**

Floatation-REST (Reduced Environmental Stimulation Therapy), an intervention that attenuates exteroceptive sensory input to the nervous system, has recently been found to reduce state anxiety across a diverse clinical sample with high levels of anxiety sensitivity (AS). To further examine this anxiolytic effect, the present study investigated the affective and physiological changes induced by Floatation-REST and assessed whether individuals with high AS experienced any alterations in their awareness for interoceptive sensation while immersed in an environment lacking exteroceptive sensation.

**METHODS:**

Using a within-subject crossover design, 31 participants with high AS were randomly assigned to undergo a 90-minute session of Floatation-REST or an exteroceptive comparison condition. Measures of self-reported affect and interoceptive awareness were collected before and after each session, and blood pressure was measured during each session.

**RESULTS:**

Relative to the comparison condition, Floatation-REST generated a significant anxiolytic effect characterized by reductions in state anxiety and muscle tension and increases in feelings of relaxation and serenity (*p* < .001 for all variables). Significant blood pressure reductions were evident throughout the float session and reached the lowest point during the diastole phase (average reduction >12 mm Hg). The float environment also significantly enhanced awareness and attention for cardiorespiratory sensations.

**CONCLUSIONS:**

Floatation-REST induced a state of relaxation and heightened interoceptive awareness in a clinical sample with high AS. The paradoxical nature of the anxiolytic effect in this sample is discussed in relation to Wolpe’s theory of reciprocal inhibition and the regulation of distress via sustained attention to present moment visceral sensations such as the breath.

Anxiety sensitivity (AS) refers to one’s fear of experiencing anxiety-related symptoms and sensations, especially those arising from within the body (1). Individuals with high AS often believe that these sensations can lead to adverse consequences, such as death, insanity, or social rejection. Such catastrophic misinterpretations make AS an anxiety amplifier; individuals with high AS are easily alarmed by anxiety-related sensations, and exposure to such sensations often further intensifies their anxiety (1). For this reason, AS has been referred to as a fundamental fear distinct from derivative ones such that the fear of anxiety can provide a motive for avoiding any stimulus likely to incite anxious symptoms (2). Consequently, most cases of chronic anxiety—including panic disorder, agoraphobia, generalized anxiety disorder, social anxiety disorder, and posttraumatic stress disorder—also feature high levels of AS, making AS a core construct underlying the initiation and maintenance of pathological anxiety (3,4).

Recent evidence suggests that reducing AS may be important for the prevention and treatment of anxiety across diagnostic categories. Prospective studies have shown that AS is a strong predictor for the onset of mood and anxiety disorders and the development of spontaneous panic attacks (1,5,6), whereas longitudinal studies have shown that individuals with high AS have a propensity for greater chronicity of illness and a higher likelihood of experiencing future anxiety symptoms (3,7,8). Controlled studies have shown significant reductions in AS following successful treatment with psychotherapy (9) or pharmacotherapy (10), and several transdiagnostic treatments have been developed to specifically target AS using different forms of interoceptive exposure (11–14). Taken together, this evidence supports the notion that AS is a fundamental driver of anxiety, and treatments that target AS have the potential of helping patients overcome anxiety regardless of their specific anxiety diagnosis (11).

Our laboratory has recently started to investigate a novel intervention for anxiety that may be beneficial for patients with high levels of AS. Referred to as Floatation-REST (Reduced Environmental Stimulation Therapy), the procedure entails floating supine in a shallow pool of water saturated with magnesium sulfate (Epsom salt). The float experience is calibrated so that sensory signals from visual, auditory, olfactory, gustatory, thermal, tactile, vestibular, gravitational, and proprioceptive channels are minimized, as are most movement and speech. Prior research investigating Floatation-REST has mostly focused on healthy populations, with the most consistent finding being decreases in indices of stress and increases in relaxation as measured from before to after the float session (15,16). Thus far, there has been only one controlled study in participants with clinical anxiety, and the findings showed significant reductions in self-reported symptoms of generalized anxiety following 12 sessions of Floatation-REST that was maintained at 6-month follow-up (17). In a recently completed open-label study (18), we recruited a sample of 50 anxious and depressed participants spanning a range of different anxiety and stress-related disorders (including posttraumatic stress disorder, generalized anxiety disorder, social anxiety disorder, panic disorder, and agoraphobia). Participants underwent a single 1-hour session of Floatation-REST, and overall the procedure was well tolerated, with no major safety concerns or adverse events. Regardless of diagnosis, the float experience induced a strong short-term reduction in state anxiety and a substantial improvement in mood. A subgroup analysis revealed that the participants with the highest AS experienced the greatest reduction in anxiety. To follow up on these findings, the current investigation recruited participants with high AS from the initial open-label study to complete a more intensive protocol that included both a comparison condition and concurrent measurement of blood pressure (BP), a key index of the relaxation response (19). Since other transdiagnostic treatments targeting AS feature manipulations that enhance exposure to interoceptive sensations (11–14), we were also interested in exploring whether the float environment altered interoceptive awareness, a construct that surprisingly has not been formally investigated in prior studies of Floatation-REST despite initial anecdotal reports of enhanced cardiac awareness (20) as well as initial experimental evidence of enhanced cardiac control (21). We hypothesized that by removing exteroceptive sensation, Floatation-REST would enhance awareness for interoceptive sensation.

## METHODS AND MATERIALS

All study procedures were approved by the Western Institutional Review Board, and all participants provided written informed consent prior to participation. The trial was registered on ClinicalTrials.gov (https://clinicaltrials.gov/show/NCT03051074), and this study is part of a larger project examining the subjective, physiological, and neural effects of Floatation-REST.

### Participant Recruitment and Randomization

The current protocol used a within-subject crossover design. Participants who met specific inclusion and exclusion criteria (see Supplemental Table S1 and Supplement) were randomly assigned (Supplemental Figure S1) to complete either a 90-minute session of Floatation-REST (referred to as the float condition) or a 90-minute session of an exteroceptive comparator (referred to as the film condition) that entailed watching a nature documentary from the BBC *Planet Earth* series (22). After completion of one condition, participants crossed over to the other condition approximately 1 week later (average time between conditions was 8 days), with both conditions scheduled to occur at the same time of day for each participant. The randomization sequence was predetermined using a 1:1 allocation ratio, and the study used an open-label design with no blinding or concealed allocation. More details about the Floatation-REST intervention and the exteroceptive comparator can be found in the Supplement.

### Measures

All self-report measurements were administered electronically to participants via an electronic tablet (Apple iPad). Survey measures were obtained using REDCap (Research Electronic Data Capture; www.project-redcap.org), a secure web-based application for electronic collection and management of research and clinical trial data. Three different types of self-report measures were administered (see Supplement for specific details about each measure): baseline measures, before and after session measures, and interoceptive measures. The baseline measures assessed each participant’s current symptoms and level of functioning during the time period of the study. The before and after measures were collected at two time points, approximately 30 minutes before and after each float or film session, to assess state-related changes in anxiety [primary outcome measure: change score on Spielberger State Anxiety Inventory (23)] and relaxation. At each time point, participants rated how they felt “right now, in the present moment.” In contrast, the interoceptive measures were aimed at gathering retrospective data about how participants felt during the actual float or film experience. Participants also completed a short debriefing interview with the experimenter at the end of the float condition to gather more qualitative information about the float experience and assess for adverse reactions. Finally, BP was measured at 10-minute intervals during each float or film session using a wireless and waterproof setup (see Supplement).

### Statistical Analysis

Change scores were computed for all before- and after-session measures, and most analyses were focused on between-session contrasts of the change scores. To be consistent with the range of scores (0–100) on the visual analog scale, each participant’s raw score for state anxiety, serenity, and interoceptive attention was first converted into standardized POMP units [representing the percent of maximum possible for each measure ranging from 0 to 100% (24)]. All measures were analyzed by linear mixed-effects models (LMMs). The LMM included fixed effects of session (float vs. film), time (after vs. before for the before and after measures; 0, 5, 10, 15, 25, 35, 45, 55, 65, and 75 minutes for the BP measures), and session-by-time interactions; a random intercept and/or a random time-slope were considered to account for participant-specific random effects. As all the interoceptive measures were focused on a single period of time (either during the float session or during the film), the LMM applied to these measures included only fixed effects of session. We focused on the session-by-time interactions for all before-session and after-session measures and BP measures, and between-session differences for all interoceptive measures. The *p* values were corrected for multiple comparisons by controlling false discovery rate at a 5% level. For each outcome measure, we also explored the potential effects of different covariates (age; sex; medication status; randomization order; and baseline severity of psychiatric symptoms based on scores from the Overall Anxiety Severity and Impairment Scale, Patient Health Questionnaire nine-item depression scale, and Sheehan Disability Scale), and we report covariates chosen by the Bayesian information criterion at the end of the Supplement. All analyses were performed with RStudio version 1.0.136 (R Foundation for Statistical Computing, Vienna, Austria) with R version 3.3.2, using the R packages *lme4* (version 1.1–14) for LMM and *lmerTest* (version 2.0–33) for calculation of degrees of freedom and *p* values based on the Kenward-Roger method.

## RESULTS

### Sample Characteristics

There were 31 participants who met inclusion and exclusion criteria (Supplemental Table S1) and underwent Floatation-REST and the exteroceptive comparison condition. All participants met criteria for one or more anxiety disorders; Table 1 provides additional details about subject demographics and baseline level of functioning. The sample spanned the spectrum of different anxiety and stress-related disorders, with a mix of comorbidities, including generalized anxiety disorder (*n* = 17), social anxiety disorder (*n* = 11), panic disorder (*n* = 9), agoraphobia (*n* = 8), and posttraumatic stress disorder (*n* = 11). Nearly every participant also had comorbid unipolar major depressive disorder (*n* = 29). Two thirds of the participants (*n* = 21) were stably medicated (for 6 weeks or longer) on one or more psychotropic medications, including selective serotonin reuptake inhibitors, serotonin and norepinephrine reuptake inhibitors, norepinephrine and dopamine reuptake inhibitors, benzodiazepines, opiates, and tricyclic antidepressants. At baseline (Table 1), most participants were acutely anxious and depressed, with average scores well above the clinical range of severity (Overall Anxiety Severity and Impairment Scale score = 10.0; Patient Health Questionnaire nine-item depression scale score = 11.6). Participants also presented with high levels of AS (average Anxiety Sensitivity Index-3 total score = 28.1) as well as marked impairment in social and occupational functioning (average total disability score on the Sheehan Disability Scale = 14.7).

### Safety and Tolerability of Interventions

There were no serious adverse events or major safety concerns arising during or after Floatation-REST. Most participants chose to float for the entire 90-minute duration, with the exception of 5 participants who exited the pool shortly after the music signaling the end of the float session started playing (approximately 85 minutes into the float). All participants completed the 90-minute film.

Overall, participants rated both conditions as being pleasant on a 100-point bipolar valence scale ranging from −50 (extremely unpleasant) to −50 (extremely pleasant). The average valence rating for the float condition was 32.1 (SD 10.8), and the average valence rating for the film condition was 21.6 (SD 18.3), with participants rating the float as significantly more pleasant than the film (*t*_30_ = 2.77, *p* < .01).

### Measures Before and After Floatation-REST

**Figure 1:**
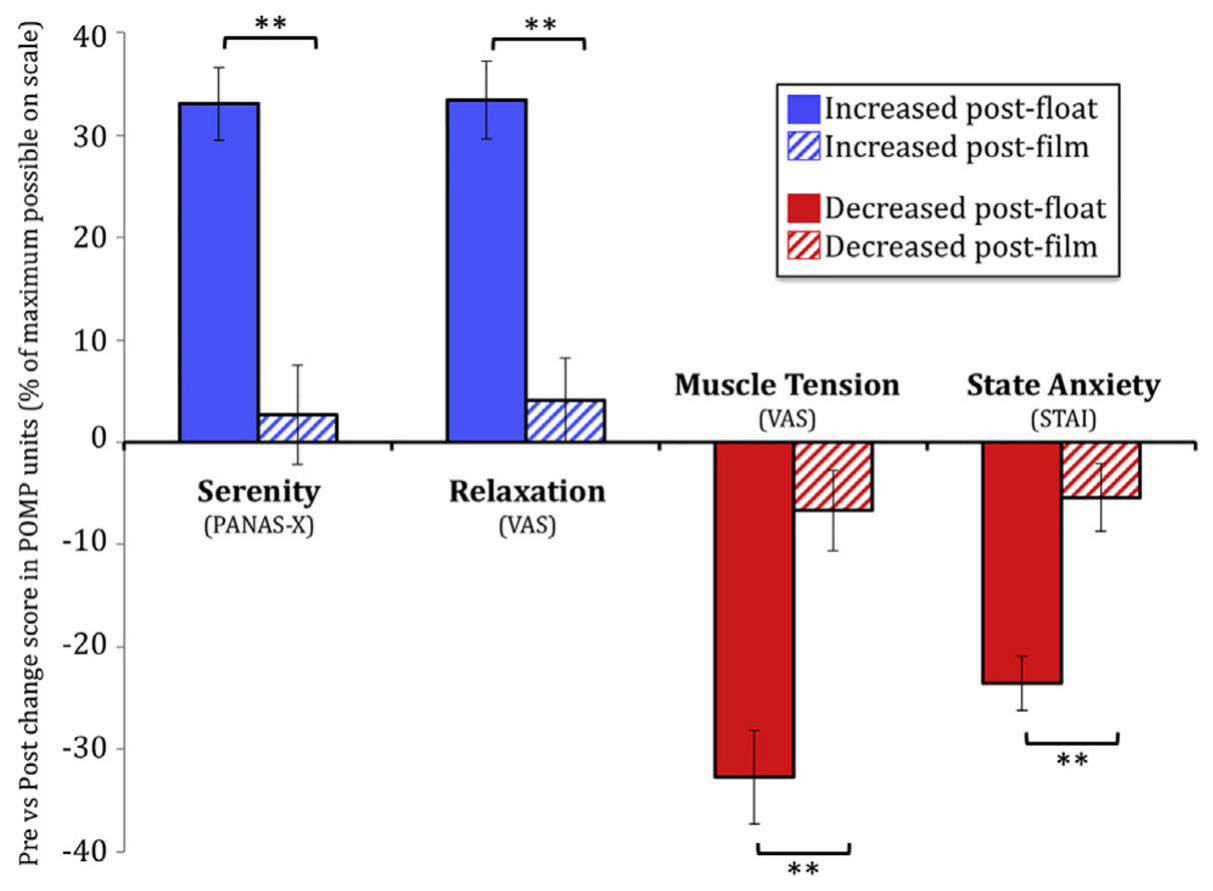
Anxiolytic effect of floatation therapy. **p < .001. Error bars represent SEM. PANAS-X, Positive and Negative Affect Schedule–Expanded Form; POMP, percent of maximum possible; STAI, State-Trait Anxiety Inventory; VAS, visual analog scale.

**Figure 2:**
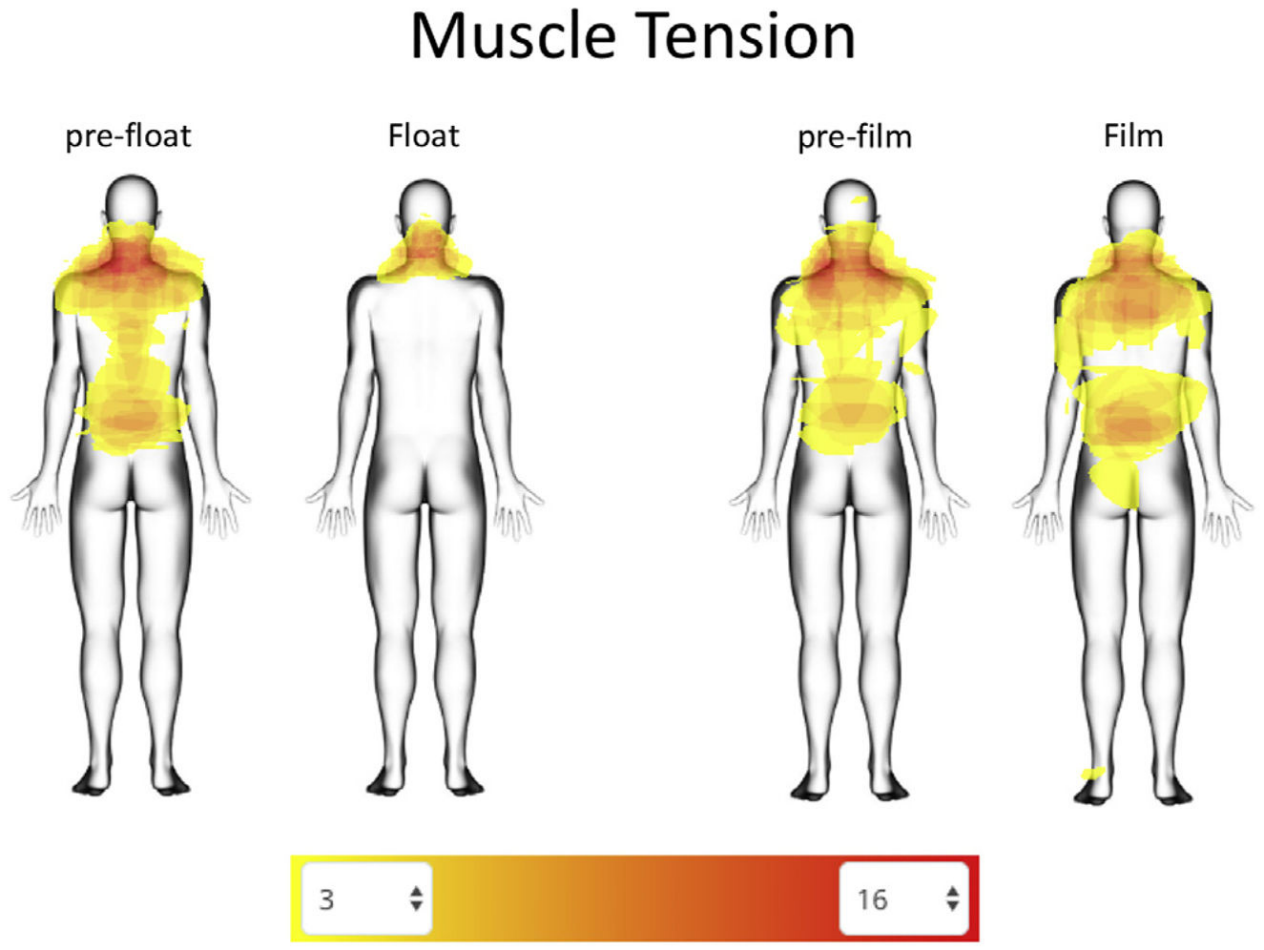
Muscle tension tracings. Participants traced any regions where they felt muscle tension. The color scale is filtered to show areas of overlap ranging from 3 to 16 participants.

All measures before and after Floatation-REST (Figure 1) showed a significant session-by-time interaction (*p* < .001). More specifically, after the float condition, participants reported substantial reduction in state anxiety and muscle tension and substantial increases in serenity and relaxation. In comparison, after the film condition, participants reported a similar direction of change on these measures, but the magnitude of change was significantly smaller than the float condition (Figure 1). Further exploration into the muscle tension changes revealed that the reduction in muscle tension while floating was felt most prominently throughout the upper and lower back (Figure 2), although some residual tension remained in the neck. In contrast, the film condition had little effect on muscle tension and seemed to elicit an increase in the number of participants reporting tension in regions of the upper and lower back and gluteus muscles (Figure 2).

### Blood Pressure

**Figure 3:**
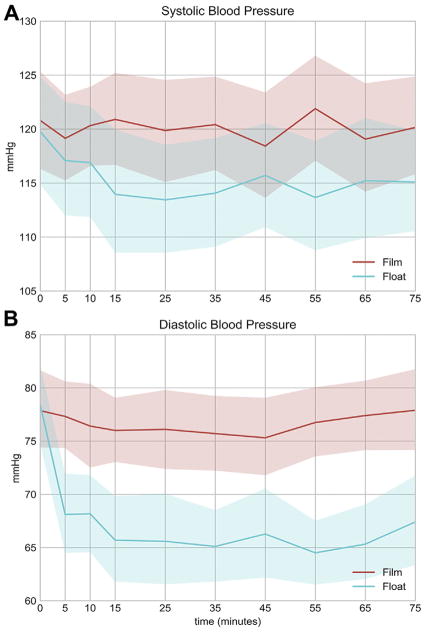
Average **(A)** systolic and **(B)** diastolic blood pressure over time in the float and film conditions. Time 0 is baseline. Shaded regions represent the pointwise 95% confidence interval.

At baseline, just before beginning the float or film, participants started at a similar level of BP (Figure 3). The float condition, but not the film, induced a reduction in both systolic BP (Figure 3A) and diastolic BP (Figure 3B). The session-by-time interaction was significant for diastolic BP (*p* < .001) but only marginally for systolic BP (*p* = 0.13). Nevertheless, there was still a highly significant main effect of session for systolic BP (*p* < .001). The drop in diastolic BP during the float session was more than twice as large as the drop in systolic BP and occurred more rapidly, evident even at the first measurement taken 5 minutes into the float session (Figure 3B). For both systolic and diastolic measures, BP reductions were most prominent over the first 15 minutes and then tended to plateau throughout the remainder of the float session. The average change from baseline, as calculated across the plateau phase of the float session (15–75 minutes), showed an overall reduction in systolic BP of 5.3 mm Hg and an overall reduction in diastolic BP of 12.8 mm Hg. The 95% confidence interval was computed at each time point that BP was measured (Figure 3), revealing a large spread between values obtained during the float versus film conditions for diastolic BP (Figure 3B). Notably, diastolic BP was reduced in every participant during the float condition. In comparison, the film condition did not significantly alter BP, with an average drop in systolic of 0.7 mm Hg and an average drop in diastolic of 1.4 mm Hg.

### Interoceptive Measures

**Figure 4:**
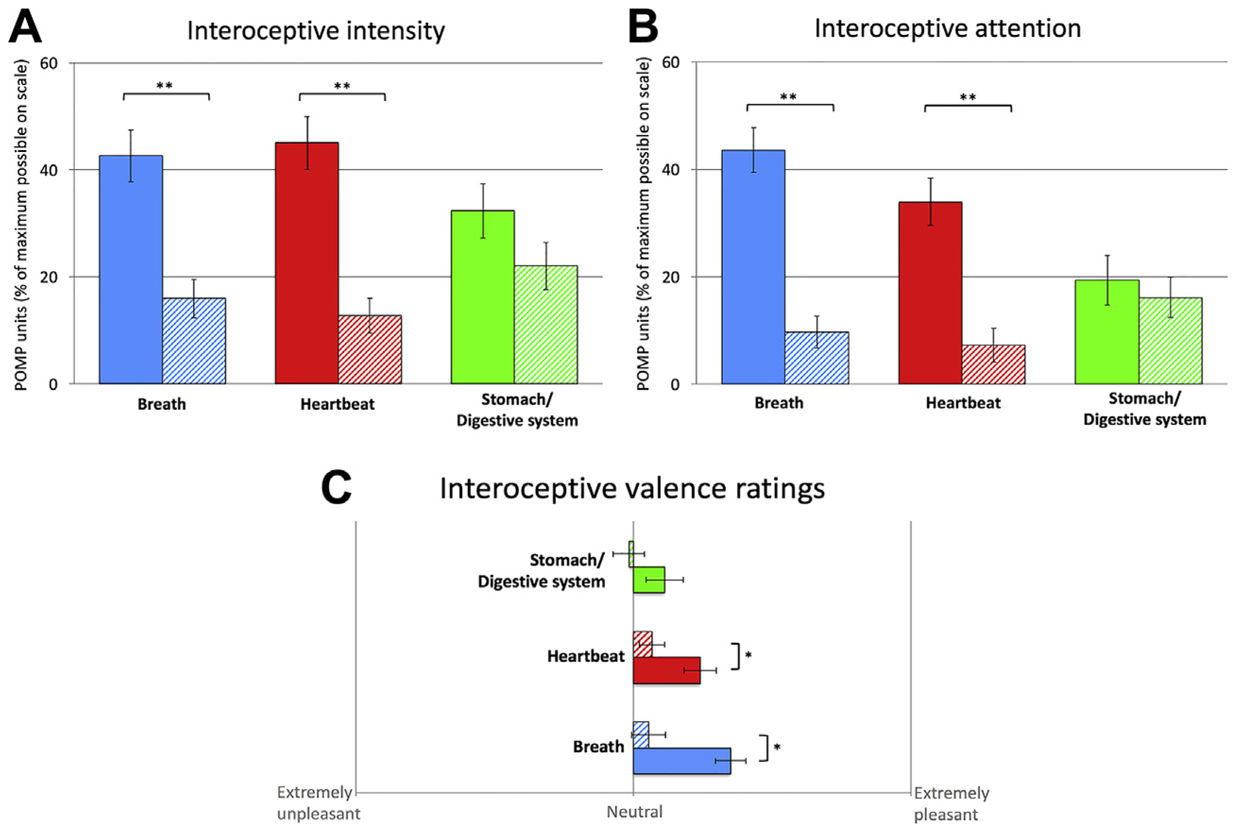
Interoception ratings during the float (solid bars) and film (hatched bars) conditions. Error bars represent SEM. Ratings of **(A)** intensity, **(B)** attention, and **(C)** valence are shown for three different visceral systems. *p < .05; **p < .001. POMP, percent of maximum possible.

**Figure 5:**
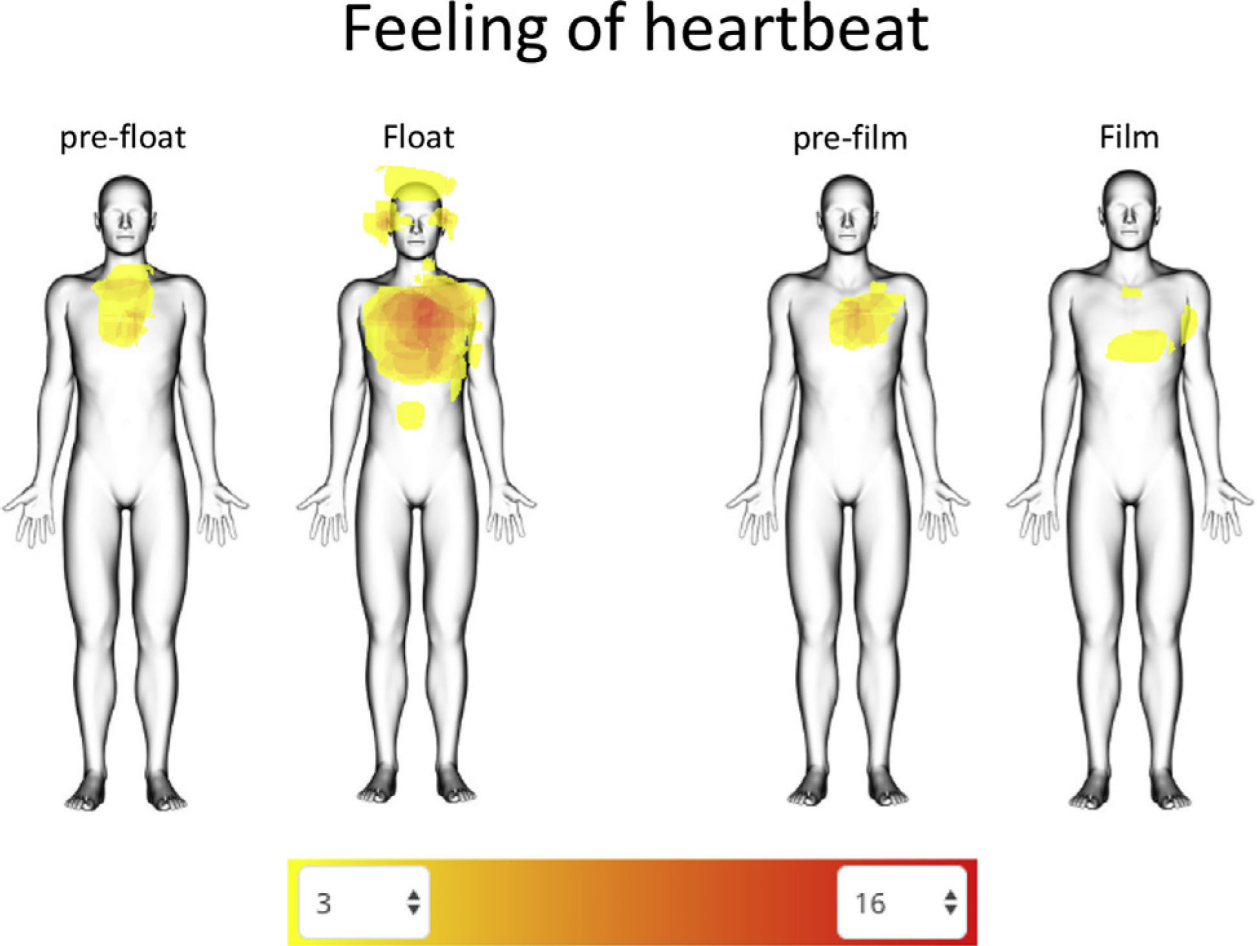
Heartbeat sensation tracings. Participants traced any regions where they felt their heartbeat. The color scale is filtered to show areas of overlap ranging from 3 to 16 participants.

**Figure 6:**
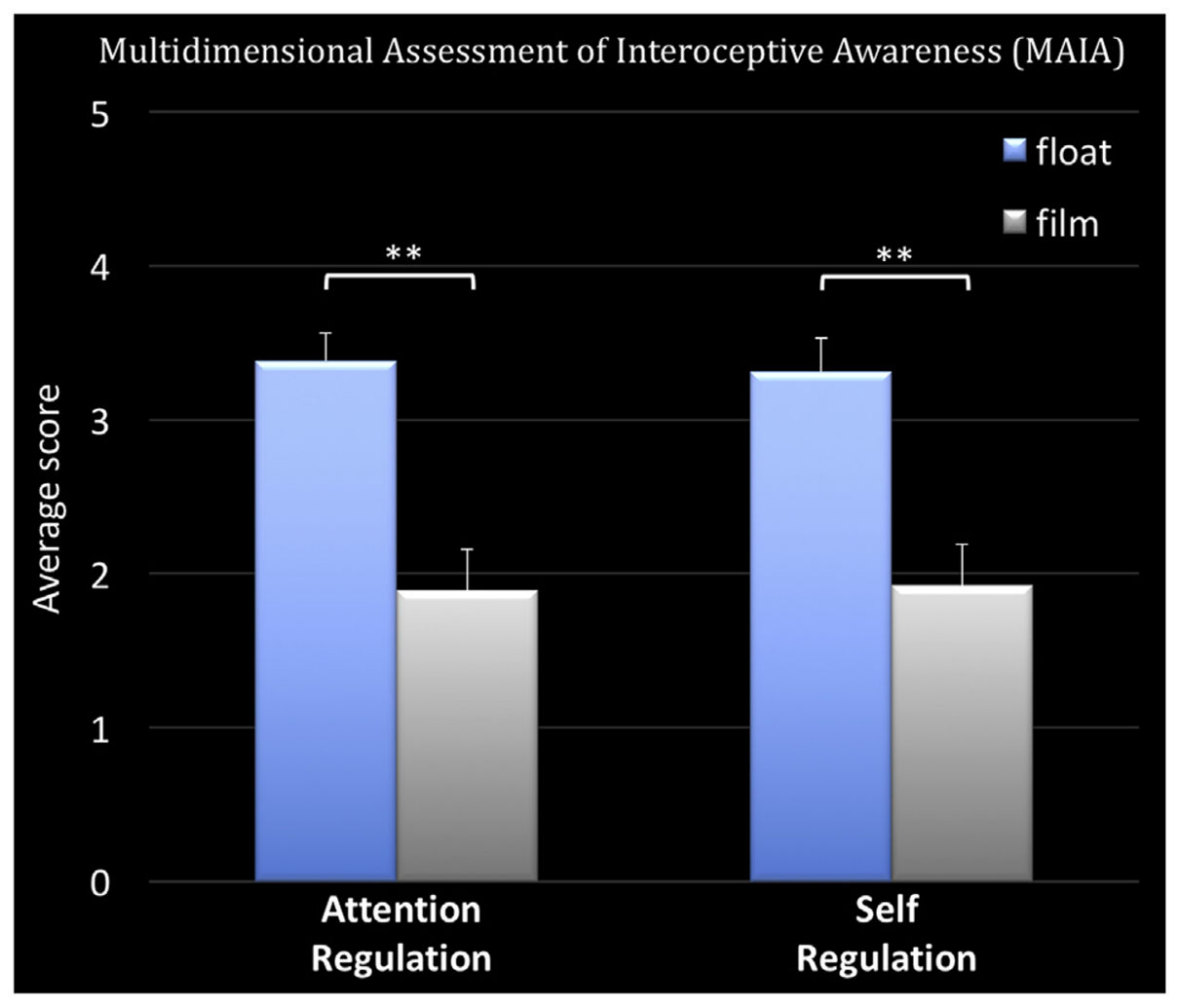
Average score (range, 0–5) on the attention regulation and self-regulation subscales of the Multidimensional Assessment of Interoceptive Awareness. Error bars represent SEM. **p < .001.

During the float session, participants reported a significant increase (*p* < .001) in the intensity of cardiorespiratory sensations compared with the film condition (Figure 4A). Likewise, participants also reported a significant increase (*p* < .001) in attention to cardiorespiratory sensations during the float session (Figure 4B) and reported that these sensations felt significantly (*p* < .05) more pleasant while floating than during the film (Figure 4C). Interestingly, the significant increases in interoceptive intensity, attention, and positive valence during the float were specific to cardiorespiratory sensations (i.e., breath and heartbeat) but not gastrointestinal sensations from the stomach and digestive system. Whereas most participants did not feel their heartbeat during the film condition, many reported a clear expansion in where the heartbeat sensation was experienced during the float session, including the chest, ears, eyes, and top of the scalp (Figure 5). On a modified state version of the Multidimensional Assessment of Interoceptive Awareness (25), floating significantly enhanced (*p* < .001) attention regulation (ability to sustain attention on body sensations) and self-regulation (ability to regulate distress by attending to body sensations such as the breath) compared with the film (Figure 6). This pattern of improved self-regulation and heightened interoceptive awareness and attention for cardiorespiratory sensations was also a common theme conveyed by participants during the postfloat debriefing (see the Debriefing Transcriptions in the Supplement).

## DISCUSSION

There were two main findings in this study: 1) a group of clinically anxious and depressed individuals with high levels of AS experienced a robust relaxation response during and after Floatation-REST that was decisively anxiolytic in nature (18); and 2) the float environment enhanced interoceptive awareness and attention to cardiorespiratory sensations. For both findings, the effects during Floatation-REST were significantly greater than during the exteroceptive comparison condition. Each of the main findings is discussed in greater detail below.

First, the float environment elicited a relaxation response that was evident both physiologically (via reduced BP) and psychologically (via reduced levels of state anxiety and muscle tension and increased levels of relaxation and serenity). Notably, the relaxation response was significantly larger during Floatation-REST than during the comparison condition, which involved an activity that many people use to help them relax—watching television (in this case a low-arousal pleasant nature documentary from the BBC *Planet Earth* series). As this was every participant’s second float session, the significant self-report changes represent a replication of the anxiolytic effect previously observed during the first float session (18), an effect that was similarly characterized by reductions in state anxiety and muscle tension and increases in relaxation and serenity. Interestingly, the magnitude of state anxiety reduction found in the current study was commensurate with the magnitude of reduction found in the initial float study (with both studies showing an average reduction of approximately 14 points when calculating the before-session to after-session change using the raw total score on the Spielberger State Anxiety Inventory) (18).

This investigation was the first study to measure BP changes during an actual session of Floatation-REST. Although the findings await further replication, they are consistent with previous research showing longer-term reductions in BP following the completion of multiple float sessions (26–29). Future work will need to closely track the temporal course of these BP fluctuations to determine how long the effects last after a float session is over. During the float session, the reduction in BP occurred over the first 15 minutes, eventually reaching a plateau that persisted for the remainder of the session. On average, systolic BP reduced by approximately 5 mm Hg, whereas diastolic BP showed a more pronounced reduction of approximately 13 mm Hg that occurred quite rapidly (often within the first 5 minutes of the float session). Potential factors contributing to the noted reductions in BP are discussed in the Supplement.

The mechanism of action underlying the physiological and psychological changes elicited by Floatation-REST is currently unknown but is likely multifaceted. For example, the reduction in state anxiety is likely a by-product of the float environment, which minimizes exposure to most external triggers of stress and anxiety, providing a chronically anxious and hypervigilant nervous system with a rare respite from the daily barrage of external triggers that it has been sensitized to over the years. The reduction in BP could be related to peripheral vasodilation caused by immersion in the warm water, possibly mediated through relaxation of vascular smooth muscles (see Supplement). Likewise, relaxation of skeletal muscles and the concomitant reduction in both muscle tension and movement is likely related to the water density (calibrated to a specific gravity of approximately 1.26 to suspend the body in a state of neutral buoyancy, where approximately half of the body is floating above the surface of the water and the other half is submerged under the water). The reduction in muscle tension, especially in the upper and lower back, was one of the more prominent effects found in this study and could play an important role in the positive benefits derived from Floatation-REST. Consistent with this notion, a recent investigation found that musculoskeletal pain was the most commonly reported somatic symptom across all types of depressive and anxiety disorders (30), and it remains possible that Floatation-REST is uniquely suited to address these somatic issues.

Second, with regard to interoception, the data suggest that being immersed in an environment lacking exteroceptive sensation does seem to alter the experience of interoceptive sensation, leading present moment visceral sensations to emerge at the center of conscious experience during Floatation-REST. This floatation-induced internal sensory enhancement appeared to show some degree of specificity for cardiorespiratory visceral sensations, whereas gastrointestinal sensations from the stomach and digestive system were not enhanced. More specifically, the float environment seemed to reflexively increase the intensity for, and attention to, interoceptive sensations related to the breath and heartbeat (Figure 4). These findings are notable, especially since most individuals, including experienced meditators, show relatively poor interoceptive awareness for cardiac sensation under resting conditions (31,32). Although individuals with high AS do tend to have heightened interoceptive awareness (33,34), it is worth emphasizing that the aforementioned enhancement effects cannot be fully attributed to a global increase in interoceptive awareness in this sample, as these same participants did not report any enhancement during the film condition. Given the heightened AS in this sample and the heightened awareness of cardiorespiratory sensations, it is notable that these sensations were rated as pleasant (Figure 4C), a finding that surprised a number of participants who were used to associating cardiorespiratory sensations with the feeling of anxiety (see Debriefing Transcriptions in the Supplement).

The current results present a paradox, as one might expect individuals with high AS to find the heightened experience of interoceptive sensations to be anxiety inducing (rather than reducing). Indeed, years of conditioning have linked interoceptive sensations to the experience of anxiety (35,36), and in the case of AS, this conditioning process can quickly go awry, triggering a pervasive pattern of avoidance that often culminates in one or more of the anxiety disorders. This brings forth the question as to why patients with high levels of AS would find serenity in an environment that enhances awareness for visceral systems previously linked to anxiety. Perhaps one answer to this paradoxical question is encapsulated by Wolpe’s seminal theory on reciprocal inhibition, premised on the notion that it is physiologically implausible for the nervous system to be in a state of anxious arousal and a state of relaxation at the same time (37). Wolpe’s theory can be summarized as follows: “If a response antagonistic to anxiety can be made to occur in the presence of anxiety-evoking stimuli so that it is accompanied by a complete or partial suppression of the anxiety responses, the bond between these stimuli and the anxiety responses will be weakened” (37, p. 71). Building on this theory, the findings presented here suggest that Floatation-REST may shift the nervous system into a physiologically quiescent state, one that is antagonistic to anxiety. At the same time, the float environment appears to enhance awareness for visceral systems intimately linked with the experience of anxiety. The emerging clinical effect, however, appears to be one of anxiety reduction, evident in individuals with a range of different anxiety and stress-related disorders who all share the common feature of AS. In cases of high AS, it is possible that part of the anxiolytic effect induced by Floatation-REST stems from reciprocal inhibition, whereby the bond between visceral sensations and anxiety is weakened, and a competing association is formed, one that links the experience of visceral sensations with a state of relaxation instead of anxiety. Thus, if Wolpe’s theory holds true, Floatation-REST may not only lead to short-term reductions in anxiety, but also, over time and with repeated exposure, the practice may lead to long-term reductions in AS.

### Limitations and Future Directions

Although this study used a within-subject randomized controlled design, replication in a larger sample, with longitudinal follow-up, and a more active comparator will be critical next steps for assessing the anxiolytic efficacy of Floatation-REST. Given that all participants completed an initial float session before this study, the current results may be affected by demand characteristics and biased responses stemming from the first float session. The film comparator used in the current study employed exteroceptive audiovisual stimulation while participants sat upright in a chair, features that likely magnified the differences between conditions on measures of interoceptive awareness and muscle tension. In addition, the current study was limited by its focus on acute effects following a single float session, and it will be imperative to explore the cumulative effects of multiple float sessions in anxious populations to determine whether there is evidence for sustained long-term benefit (17) or signs of adverse effects. In addition, very little is known about how long the acute effects persist after a float is over, and a better understanding for the duration of the acute effects will help determine other factors, such as the optimal “dose” and frequency of floating. Beyond BP, it will be important to also explore other physiological (e.g., heart rate variability and respiration) and neural (e.g., electroencephalography and/or functional magnetic resonance imaging) parameters to have a more complete understanding of how Floatation-REST affects the nervous system. Likewise, the interoceptive measures in the current study were derived via self-report, and future studies should employ behavioral paradigms for more objectively assessing interoceptive accuracy.

The enhancement of awareness and attention for cardio-respiratory sensations during Floatation-REST occurred without explicit mindfulness instruction or training. The seemingly reflexive nature of the interoceptive enhancement provided by the float environment may have important therapeutic implications, as the cultivation of present moment awareness via sustained attention to the breath is a fundamental feature of many meditative traditions (38,39). Although the practice of meditation appears to help reduce anxiety (40), the effects are often of a small to medium size (41,42), with many acutely anxious patients finding it difficult to sustain their focus on present moment sensations (43). In this light, the float environment may help anxious individuals anchor their attention onto internal sensations such as the breath, both by extreme filtering of all external sensory distractors and stressors and by enhancing the feeling of the heartbeat and the breath. The data further suggest that Floatation-REST may help bolster self-regulation and the reduction of anxiety and distress through sustained attentional focus on present moment body sensations (Figure 6), highlighting the conducive nature of the float environment for facilitating the learning of core skills involved in the training of mindfulness (44). Future research should further explore these preliminary findings to determine whether Floatation-REST facilitates the practice of mindfulness and whether the combination of floating with specific mindfulness instructions can lead to even greater anxiolytic effects.

## Supplementary Material

supplement

## Figures and Tables

**Table 1:** Participant Demographics and Baseline Functioning

Variable	All Participants	Participants First Randomized to Film	Participants First Randomized to Float
Sample Size	31	16	15
Age, Years	39.1 (11.1)	38.2 (11.7)	39.9 (11.0)
Sex, Male/Female	12/19	4/12	8/7
Medicated Subjects	21	9	12
Anxiety Sensitivity (ASI-3)	28.1 (12.3) [23.6, 32.6]	29.4 (14.2) [21.5, 37.3]	26.9 (10.6) [21.2, 32.5]
Anxiety Severity (OASIS)	10.0 (3.8) [8.6, 11.4]	9.5 (4.3) [7.2, 11.9]	10.4 (3.4) [8.6, 12.3]
Depression Severity (PHQ-9)	11.6 (5.5) [9.6, 13.6]	9.9 (5.8) [6.7, 13.2]	13.1 (4.8) [10.5, 15.7]
Level of Disability (SDS)	14.7 (8.0) [11.8, 17.6]	13.2 (8.1) [8.7, 17.7]	16.1 (7.8) [12.0, 20.3]

The total or average scores are presented for each metric. Numbers inside parentheses represent the standard deviation, and numbers inside brackets represent the 95% confidence interval.

ASI-3, Anxiety Sensitivity Index-3; OASIS, Overall Anxiety Severity and Impairment Scale; PHQ-9, Patient Health Questionnaire nine-item depression scale; SDS, Sheehan Disability Scale.
