# Supplementary material for: The Elicitation of Relaxation and Interoceptive Awareness Using Floatation Therapy in Individuals With High Anxiety Sensitivity
Source: Biol Psychiatry Cogn Neurosci Neuroimaging. Author manuscript; Available in PMC 2018 Jul 11. (PMC6040829; doi:10.1016/j.bpsc.2018.02.005)
Supplement: supplement [file NIHMS973242-supplement.pdf]

The Elicitation of Relaxation and Interoceptive Awareness Using Floatation Therapy in Individuals with High Anxiety Sensitivity

Supplemental Information

Participant Recruitment

All participants were recruited from a previous study where they underwent a single 60-minute float session (without any physiological measurements) to help acclimate them to the float environment (1). On average, there was a 2-month gap between completing the previous study and the current study. In the previous study, participants were recruited through the Tulsa 1000 (T1000) database maintained at the Laureate Institute for Brain Research (LIBR) using specific inclusion and exclusion criteria (Table S1). The T1000 is a naturalistic study that aims to recruit and longitudinally follow 1000 treatment-seeking individuals from the local community, many of whom have anxiety and/or depression (2). Each participant received the Mini-International Neuropsychiatric Interview (MINI) version 6.0 (3), and all psychiatric diagnoses were confirmed following review of the clinical history by a board-certified psychiatrist.

The specific inclusion and exclusion criteria (Table S1) for recruiting participants from the T1000 database into the initial float study targeted individuals with very high levels of AS (defined as an Anxiety Sensitivity Index (ASI-3) total score ≥ 30) across the spectrum of different anxiety and stress-related disorders, many with comorbid unipolar depression. Participants were invited back to participate in the current study so long as they continued to present with at least a mild to moderate degree of anxiety severity during the initial float study, defined as an Overall Anxiety Severity and Impairment Scale (OASIS) score ≥ 6. From the original 50 participants in the initial float study, 43 were

invited to participate in the current study (Figure S1) which took place during the first half of 2017. Both the ASI-3 and OASIS were re-administered at the start of this study, and the updated scores are presented in Table 1. Since the T1000 is a naturalistic study based on a community sample, we allowed participants who were stably medicated into the study. However, we added exclusion criteria for more severe forms of psychopathology and substance use in order to minimize potential safety risks.

Table S1. Inclusion and Exclusion Criteria for the Initial Float Study

Inclusion Criteria	Exclusion Criteria
1. DSM-IV diagnosis on the MINI of an Anxiety Disorder (Generalized Anxiety Disorder, Social Anxiety Disorder, Panic Disorder, Agoraphobia) and/or Posttraumatic Stress Disorder (PTSD)<br>2. Overall Anxiety Severity and Impairment Scale (OASIS) score ≥ 8<br>3. Anxiety Sensitivity Index (ASI-3) total score ≥ 30<br>4. If taking medication, must be stably medicated prior to participation (defined as having taken the medication for 6 weeks or longer)<br>5. Between 18-55 years of age<br>6. No prior Floatation-REST experience	1. Comorbid Bipolar Disorder or Schizophrenia<br>2. Active suicidality with intent or plan<br>3. Currently receiving inpatient treatment<br>4. Current Substance Use Disorder ≥ moderate<br>5. History of neurological conditions (e.g., epilepsy, stroke, severe traumatic brain injury, Parkinson's disease, Alzheimer's disease or other forms of dementia)<br>6. Any skin conditions or open wounds that could cause pain when exposed to saltwater<br>7. Inability to swim or lay comfortably in a shallow pool of water

Floatation-REST Intervention

All float sessions occurred in an open circular fiberglass pool custom-designed for research purposes by Floataway (Norfolk, United Kingdom). The open circular float pool was 8 feet in diameter and contained 11 inches of reverse osmosis water saturated with ~1,800 pounds of USP grade Epsom salt (magnesium sulfate), creating a dense salt

water solution maintained at a specific gravity of ~1.26, allowing participants to effortlessly float on their back. Since the pool had no enclosure, participants could freely enter and exit at any time. The room around the pool was constructed to be waterproof, soundproof, lightproof, and temperature-controlled (described in greater detail below). Silent heaters were placed under the pool to maintain the water at a constant temperature and a dedicated heating, ventilation, and air conditioning system maintained the air at a constant temperature. The temperature of the water and air approximated the surface temperature of the skin (~95.0°F), and could be adjusted remotely by the experimenter in a nearby control room. An intercom system allowed the participant to freely communicate with the experimenter throughout the float session should any issues arise, and specialized speakers placed around the perimeter of the pool allowed the experimenter to communicate with the participant and play music to signal the end of the session.

The float pool and surrounding room were specially engineered to minimize all sensory signals from visual, auditory, olfactory, gustatory, thermal, tactile, vestibular, gravitational and proprioceptive channels. Visual stimulation was minimized by building an entry door and gasket system which expunged all sources of outside light. In addition, there were no windows inside the float room, and the adjacent room contained a private bathroom that also had no windows, and no lights (which were automatically shut off during the float itself). Thus, when the entry door to the float room was sealed and the blue LED light inside the pool was turned off, the float room was completely dark. Auditory stimulation was minimized by constructing the float room using multiple layers of sound dampening walls with thick insulation and added soundproofing material, restricting most outside airborne sound from entering the room. Structural sounds transmitted via

vibrations in the floor were minimized by having the float pool rest on a bed of 48 butyl rubber springs, effectively isolating the pool from the building and preventing structure-borne noises from entering the water. Olfactory stimulation was minimized by using only unscented cleaning products and having the participant shower beforehand to help remove body odors. In addition, the water disinfection system used a combination of ultraviolet light and 35% hydrogen peroxide which does not emit any odors during the oxidative process. Gustatory stimulation was minimized by having participants eat several hours before the float, while refraining from eating and drinking during the float. Thermal stimulation was minimized by setting the temperature of the water and the air to closely match the temperature at the surface of the skin, which is typically a few degrees cooler than core body temperature. All temperature sensors were calibrated using a Thermoworks precision thermometer (Utah, USA) certified by the National Institute of Standards and Technology (NIST). Throughout each float session, the water temperature was maintained at 95.0°F (±0.3°F) and the air temperature at the rim of the pool was maintained at 93.5°F (±0.5°F), slightly lower than the water temperature based on the relative humidity in the air. This temperature setting helped minimize the need for thermoregulation while reducing the perceptual boundary between air, body, and water, a unique feature of the float experience. Specific gravity of the water was calibrated using an H-B Instrument Polycarbonate Hydrometer (Pennsylvania, USA), with a specific gravity range of 1.20-1.42 and NIST calibrated to achieve an accuracy within 0.002. The density of the water and salt concentration was maintained at a specific gravity between 1.25-1.26 for all float sessions. The body's immersion in this dense saline solution minimized stimulation from tactile, vestibular, gravitational, and proprioceptive channels

by buffering the body against the forces of gravity and allowing the individual to effortlessly float on their back in a state of stillness. The importance of “stillness” was also emphasized during the pre-float instruction set, further helping to minimize both movement and speech.

All participants were instructed that they could float “for up to 90 minutes” and could stop floating at any time. The following script was read prior to the float session: “*Throughout the day, our brain and body are constantly bombarded by sensory information from the external world. In this study, we aim to understand what happens when you get a chance to disconnect from this constant stimulation by floating in an environment with reduced levels of light and sound, and reduced pressure on the spinal cord. While floating, try to find a place of stillness of both body and mind. You have complete control throughout the experience and can stop at any time. During the float we encourage you stay awake and when the float is over we will turn on some music for you. There is no rush, so please take your time exiting the pool.*” No additional instructions were provided for how participants should spend their time during the float session.

Exteroceptive Comparison Condition

The subjective and physiological changes during Floatation-REST were directly compared to an exteroceptive comparison condition that took place at the same time of day as the float session, and entailed a 90-minute period of audiovisual stimulation that matched the duration of the float session. Three episodes (*Great Plains, Jungles,* and *Seasonal Forests*) from the nature documentary Planet Earth (4) were edited into a single film clip. The film contained pleasant and serene scenes of geographic landscapes and

wildlife. Segments that had the potential to elicit intense emotional or physiological responses, such as depictions of violence or mating, were intentionally excluded. The film was presented on a computer monitor (21.5 x 15 inches) with speakers, and participants were seated upright in a stationary chair approximately 30 inches away from the monitor. As in the Floatation-REST condition, participants were encouraged to stay awake during the film and to try to find a place of stillness in order to minimize both movement and speech.

Baseline Measures

Anxiety Sensitivity Index (ASI-3): The ASI-3 is an 18-item questionnaire that has been shown to have good reliability and validity, and improved psychometric properties over the original measures (5). Questions are answered using a 4-point scale and total ASI scores can range from 0 to 72. Normative data collected in a large sample (n=4,720) of healthy North Americans indicate a mean ASI-3 total score of 12.8 (SD=10.6) (5). A meta-analysis found that patients with anxiety and depression commonly have a total ASI score around 30 (6).

Overall Anxiety Severity and Impairment Scale (OASIS): The OASIS is a 5-item questionnaire that can be used across the different anxiety disorders as a continuous measure of anxiety severity and impairment over the past week (7). Each item is rated on a 5-point scale and the ratings are summed to obtain a total score ranging from 0 to 20. A cut-score of 8 has been shown to correctly classify 87% of individuals as having a current anxiety diagnosis (8).

Patient Health Questionnaire (PHQ-9): The PHQ-9 is a 9-item measure for assessing the severity of depressive symptoms over the past 2 weeks (9). Scores of 1-4 are considered indicative of minimal depression, 5-9 mild depression, 10-14 moderate depression, 15-19 moderately severe depression, and 20-27 severe depression.

Sheehan Disability Scale (SDS): The SDS assesses how much the respondent's mental health issues are perceived to have affected their daily activities in three functional domains: work/school, social/leisure activities, and family life/home responsibilities (10). Total disability scores range between 0 to 30, with scores ≥ 5 signifying impairment (11). A review of studies using this measure indicated significant impairment in functioning in patients with anxiety disorders, who have mean total disability scores typically ranging between 14-18 (12).

Pre/Post-Measures

Primary outcome measure — State-Trait Anxiety Inventory (STAI-Y State form): The Spielberger State Anxiety Inventory is a widely used 20-item self-report questionnaire designed to assess an individual's level of anxiety at the present moment with total scores ranging from 20-80 (13). The items assess for the presence or absence of current anxiety symptoms, and the measure has been shown to have excellent internal consistency and good convergent and discriminant validity (13).

Serenity scale on the Positive and Negative Affect Schedule - Expanded Form (PANAS-X): The PANAS-X is one of the most commonly used measures of mood, with high internal consistency, and good convergent, discriminant, and construct validity (14, 15). The serenity scale on the PANAS-X has participants rate how *calm*, *relaxed*,

and *at ease* they feel at the present moment using a 5-point Likert-type response scale, ranging from 1 (Very slightly or not at all) to 5 (extremely).

Visual Analogue Scales (VAS): Participants completed several VAS measures where they rated how they currently felt on a 100-point scale that went from 0 (Not at all/None) to 100 (Extremely/The most I have ever felt). Each scale contained a digital slider (always starting at 0) that participants could move along a horizontal axis. The **Relaxation VAS** asked, “How relaxed do you feel right now?” The **Muscle Tension VAS** asked, “How much muscle tension or tightness do you feel right now?” In addition, during the post-float/film period, participants completed a 100-point bipolar valence scale asking, “Overall, how was your float experience?” or “Overall, how was the film?” The scale went from -50 (Extremely Unpleasant) to +50 (Extremely Pleasant), with the slider starting in the middle of the scale at 0 (Neutral).

Interoceptive Measures

As a first step toward assessing changes in interoceptive awareness, each participant completed a series of self-report measures during the post-float and post-film period. The questions separately probed 3 different visceral sensations (breath, heartbeat, and stomach/digestive system), where participants provided retrospective ratings of intensity, attention, and valence. The interoceptive intensity question (“How intensely did you feel your [insert visceral sensation] while floating/watching the film?) used a VAS ranging from 0 (Not at all/None) to 100 (Extremely/The most I have ever felt). The interoceptive attention question (“During today's float/film, how often was your attention focused on your [insert visceral sensation]?”) used a Likert-type response scale,

ranging from 1 (Very slightly or not at all) to 5 (The entire time). The interoceptive valence question (“How did your [insert visceral sensation] feel while floating/during the film?”) used a 100-point bipolar scale that went from -50 (Extremely Unpleasant) to +50 (Extremely Pleasant), with the slider starting in the middle of the scale at 0 (Neutral).

Participants also completed a modified version of the Multidimensional Assessment of Interoceptive Awareness (MAIA) (16), adapted with permission from Dr. Wolf Mehling. A recent study used the MAIA to assess for longitudinal changes in interoceptive awareness following 3-months of meditation training involving mindful attention to the breath and other body sensations, and found that the largest improvements in interoceptive awareness occurred on the attention regulation and self-regulation scales of the MAIA (17). In order to minimize measurement burden and focus on the dimensions of interoceptive awareness most relevant to mindfulness training, the current study had participants complete a state version of the attention regulation and self-regulation scales of the MAIA where they were asked to “indicate how often each statement applied to you during the float/film” using the same rating scale as the original MAIA ranging from 0 (Never) to 5 (Always). The attention regulation scale was comprised of 6 questions assessing one’s ability to sustain and control attention to body sensations (e.g., "I could pay attention to my breath without being distracted by things happening around me"). The self-regulation scale was comprised of 4 questions assessing one’s ability to regulate distress by attention to body sensations (e.g., "When I was caught up in thoughts, I could calm my mind by focusing on my body/breathing"). Both scales used the same items as the original MAIA, with the exception of item 13 from the attention regulation scale (“When I am in a conversation with someone, I can pay attention to my

posture”), which was removed since the question pertained to a social experience that was not part of either the float or film condition. An average score for each scale was computed by averaging the ratings from each question.

In order to obtain more qualitative information about the interoceptive changes, participants used a tablet to trace where they topographically felt their heartbeat sensation and muscle tension on a human manikin. This “Somatomap” application has been used in prior publications (18) and was created using the Chorus platform (chorus.semel.ucla.edu), a secure, web-based toolbox that enables individuals to create interactive mobile applications using a simple, visual interface (19). Tracings were provided both before (“Over the past hour, where did you feel your heartbeat?” or “Over the past hour, please draw anywhere that you felt any muscle tension or tightness?”) and after each session (“During the float/film, where did you feel your heartbeat?” or “During the float/film, please draw anywhere that you felt any muscle tension or tightness?”). An overlap heat map for the entire group was generated at each time point to show the distribution of the felt sensation across the body.

At the end of the float portion of the study, each participant completed a short debriefing interview where they were asked, “What was it like to experience internal body sensations like your breath and heartbeat?” All responses were recorded with a digital audio recorder and later transcribed. Due to technical difficulties, one participant’s response was missing. The debriefing transcriptions for the remaining 30 participants can be found below.

Blood Pressure (BP) Measurement

In addition to the self-report measures, the current study also measured BP using a QardioArm wireless BP monitor (Qardio Inc., San Francisco, California, USA), an FDA-cleared automated sphygmomanometer which uses the Oscillometric method to achieve a measurement range of 40-250 mmHg and an accuracy of ±3 mmHg. The QardioArm has been clinically validated according to ANSI/AAMI/ISO 81060-2:2009 as well as the European Society for Hypertension International Protocol Revision 2010 (20). The BP cuff was positioned approximately 1 inch above the elbow, around the left upper arm, so that it was situated at the same level as the heart. During both conditions, participants were instructed to keep their arms positioned downwards, resting along the side of their body. A LimbO Waterproof Protector (Limbo USA, Portland, Maine, USA) was placed over the BP device in order to prevent water from reaching the QardioArm during the float session. To ensure comparability across conditions, participants also wore the LimbO during the comparison condition. All BP data was wirelessly transmitted in real-time via Bluetooth 4.0 to an iPad tablet located in the adjacent control room. Each BP measurement took 30-60 seconds to complete and was initiated remotely by the experimenter using an application on the iPad. A baseline BP measurement was collected in the seated position following 5 minutes of rest immediately before starting the float/film. Nine additional BP measurements were collected once every 10 minutes during the float/film. Of note, the first 3 measurements acquired during the float/film were taken every 5 minutes in order to provide a higher temporal resolution for any potential BP changes occurring toward the beginning of a session. A small number of measurements (<3% of the total number of measurements) were either missing (due to a temporary loss in

Bluetooth connectivity with the QardioArm) or were excluded if the reading was deemed an outlier based on previous guidelines (21) that recommended excluding all measurements greater than 2 standard deviations from an individual's average BP during the float or film session. In addition, the dataset from one participant's float session and another participant's film session failed to be collected due to a complete loss in Bluetooth connectivity with the QardioArm, leaving 29 complete datasets where every participant had BP data successfully collected in both conditions.

Potential Factors Affecting BP

One potential explanation for the reduction in BP during Floatation-REST could be related to vasodilation caused by the warm 95°F environment. Indeed, a previous study showed a significant reduction in systolic (but not diastolic) BP when sitting in a room heated to 95°F (22), whereas immersion in 97°F water caused significant reductions in both systolic and diastolic BP (23). In contrast, a small sample of subjects immersed in 93°F Dead Sea water (with a specific gravity of 1.19) actually showed a significant increase in BP (24). Another possibility is related to the high quantity of Epsom salt (magnesium sulfate), which might exert an antihypertensive effect if any of the magnesium were to be transdermally absorbed through the water (25). Body position (seated during the film and supine during the float) is another factor that may be contributing to the BP changes, although prior studies directly comparing BP while supine versus sitting have found contradictory results, with some studies showing lower BP while supine (26, 27) and other studies actually showing higher BP while supine (28-30). It will

be important for future studies to ascertain the specific mechanism(s) by which Floatation-REST reduces BP.

Covariates Chosen by the Bayesian Information Criterion (BIC)

For the Pre/Post-measures, in addition to the Session-by-Time interactions, participants who were first randomized to the Film reported higher serenity (beta = 11.6, $t(33.1) = 2.478$, $p = 0.018$), and participants with higher baseline PHQ-9 scores reported more muscle tension (beta = 1.7, $t(49.0) = 3.996$, $p < 0.001$), but neither effect confounded the Session-by-Time interaction (0.0% change in both beta weights). For the interoceptive measures, in addition to the Session effects, participants who were first randomized to the Float condition perceived lower stomach intensity (beta = -16.1, $t(29.0) = -2.292$, $p = 0.029$) and breath attention (beta = -0.6, $t(29.0) = -2.889$, $p = 0.007$). Again, neither variable confounded the Session effects (0.0% changes in beta weights). For blood pressure, no covariates appeared to be associated with the Session or Session-by-Time interactions. No other covariates (age, sex, medication status, randomization order, and baseline severity of psychiatric symptoms) were selected by the BIC.

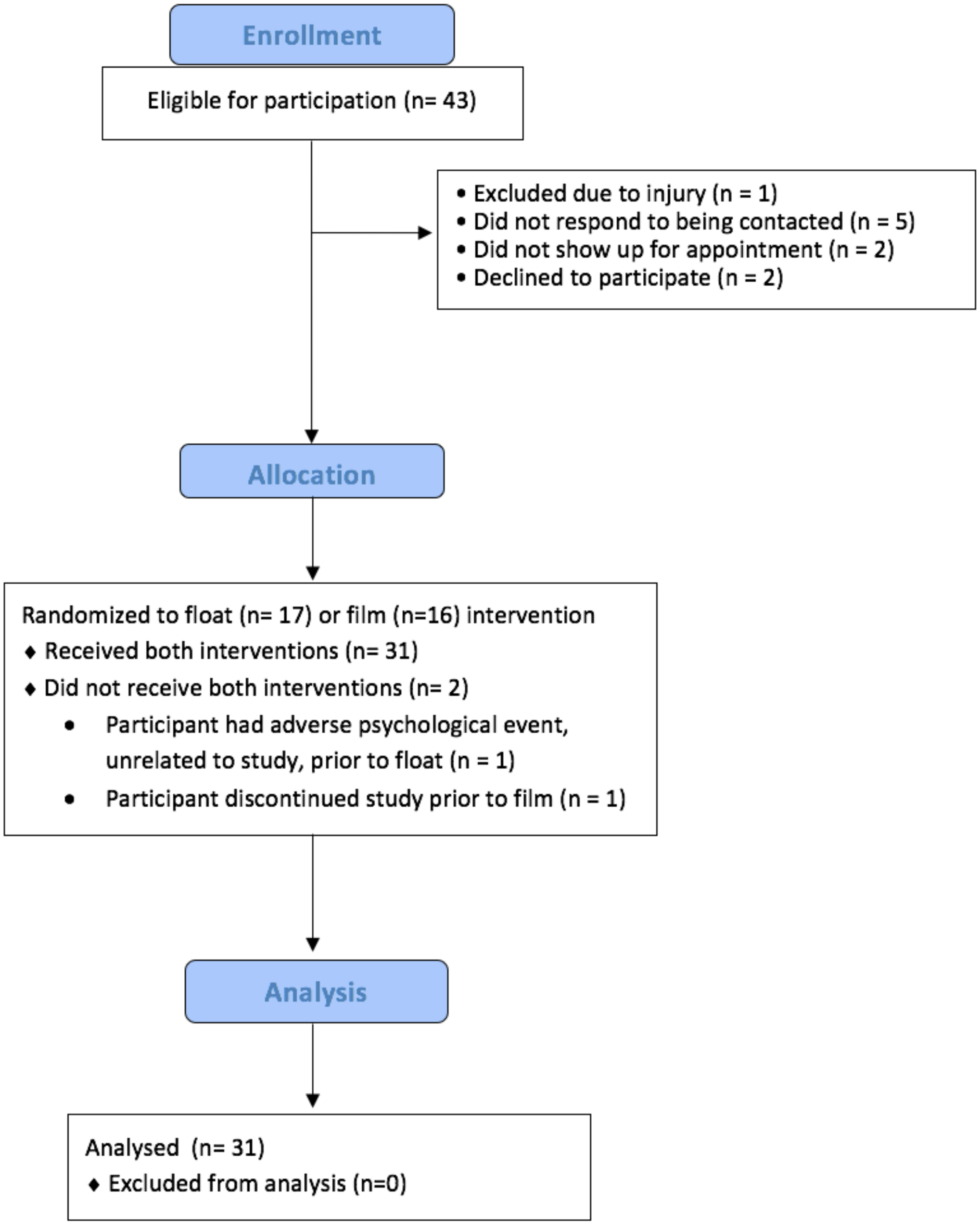

Figure S1. Flow diagram for study recruitment

Supplemental References

1. Feinstein JS, Khalsa SS, Yeh H, Wohlrab C, Simmons WK, Stein MB, et al. (2018): Examining the short-term anxiolytic and antidepressant effect of Floatation-REST. *PLoS ONE* 13:e0190292.

2. Victor TA KS, Simmons WK, Feinstein JS, Savitz J, Aupperle RL, Yeh H, Bodurka J, Paulus MP (2018): The Tulsa 1000: A naturalistic study protocol for multi-level assessment and outcome prediction in a large psychiatric sample. *BMJ Open* 8:e016620.

3. Sheehan D, Lecrubier Y, Sheehan K, Amorim P, Janavs J, Weiller E (1998): The Mini International Neuropsychi-(1998). The Mini International Neuropsychiatric Interview (MINI): The development and validation of a structured diagnostic psychiatric interview for DSM-IV and CID-10. *Journal of Clinical Psychiatry* 59:22-33.

4. Attenborough D, Fothergill A (2006): Planet Earth: complete BBC Series. *London: British Broadcasting Corporation [DVD Box Set: 5 discs]*.

5. Taylor S, Zvolensky MJ, Cox BJ, Deacon B, Heimberg RG, Ledley DR, et al. (2007): Robust dimensions of anxiety sensitivity: development and initial validation of the Anxiety Sensitivity Index-3. *Psychological assessment* 19:176-188.

6. Naragon-Gainey K (2010): Meta-analysis of the relations of anxiety sensitivity to the depressive and anxiety disorders. *Psychol Bull* 136:128-150.

7. Norman SB, Hami Cissell S, Means-Christensen AJ, Stein MB (2006): Development and validation of an overall anxiety severity and impairment scale (OASIS). *Depression and anxiety* 23:245-249.

8. Campbell-Sills L, Norman SB, Craske MG, Sullivan G, Lang AJ, Chavira DA, et al. (2009): Validation of a brief measure of anxiety-related severity and impairment: the Overall Anxiety Severity and Impairment Scale (OASIS). *Journal of affective disorders* 112:92-101.

9. Kroenke K, Spitzer RL, Williams JB (2001): The phq-9. *Journal of general internal medicine* 16:606-613.

10. Sheehan D (1983): Sheehan disability scale. *Handbook of psychiatric measures*:113-115.

11. Leon AC, Olfson M, Portera L, Farber L, Sheehan DV (1997): Assessing psychiatric impairment in primary care with the Sheehan Disability Scale. *The international journal of psychiatry in medicine* 27:93-105.

12. Sheehan KH, Sheehan DV (2008): Assessing treatment effects in clinical trials with the discan metric of the Sheehan Disability Scale. *International clinical psychopharmacology* 23:70-83.

13. Spielberger CD, Gorsuch RL, Lushene R, Vagg PR, Jacobs G (1983): *State-trait anxiety inventory for adults*. Mind Garden.

14. Watson D, Clark LA, Tellegen A (1988): Development and validation of brief measures of positive and negative affect: the PANAS scales. *Journal of personality and social psychology* 54:1063-1070.

15. Watson D, Clark LA (1999): The PANAS-X: Manual for the positive and negative affect schedule-expanded form.

16. Mehling WE, Price C, Daubenmier JJ, Acree M, Bartmess E, Stewart A (2012): The multidimensional assessment of interoceptive awareness (MAIA). *PloS ONE* 7:e48230.

17. Bornemann B, Herbert BM, Mehling WE, Singer T (2015): Differential changes in self-reported aspects of interoceptive awareness through 3 months of contemplative training. *Frontiers in psychology* 5:1504.

18. Hassanpour MS, Yan L, Wang DJ, Lapidus RC, Arevian AC, Simmons WK, et al. (2016): How the heart speaks to the brain: neural activity during cardiorespiratory interoceptive stimulation. *Phil Trans R Soc B* 371:20160017.

19. Arevian AC, O'Hora J, Jones F, Jones A, Booker-Vaughns J, Banner D, et al. (in press): Participatory Technology Development to Enhance Community Resilience. *Ethnicity & Disease*.

20. O'brien E, Atkins N, Stergiou G, Karpettas N, Parati G, Asmar R, et al. (2010): European Society of Hypertension International Protocol revision 2010 for the validation of blood pressure measuring devices in adults. *Blood pressure monitoring* 15:23-38.

21. Lee D, Farmer A, Swift C, Jackson S (1995): Investigation of ambulatory blood pressure monitoring data editing criteria. *Journal of human hypertension* 9:195-198.

22. Sollers JJ, Sanford TA, Nabors-Oberg R, Anderson CA, Thayer JF (2002): Examining changes in HRV in response to varying ambient temperature. *IEEE engineering in medicine and biology magazine* 21:30-34.

23. Hildenbrand K, Becker BE, Whitcomb R, Sanders JP (2010): Age-Dependent Autonomic changes Following immersion in cool, neutral, and warm water Temperatures. *International Journal of Aquatic Research and Education* 4:127-146.

24. Ish-Shalom N, Better O (1984): Volume regulation in man during neck-out immersion in a medium with high specific gravity (Dead Sea water). *Israel journal of medical sciences* 20:109-112.

25. Zhang X, Li Y, Del Gobbo LC, Rosanoff A, Wang J, Zhang W, et al. (2016): Effects of Magnesium Supplementation on Blood Pressure: A Meta-Analysis of Randomized

Double-Blind Placebo Controlled Trials. *Hypertension*:HYPERTENSIONAHA. 116.07664.

26. Cicolini G, Pizzi C, Palma E, Bucci M, Schioppa F, Mezzetti A, et al. (2011): Differences in blood pressure by body position (supine, Fowler's, and sitting) in hypertensive subjects. *American journal of hypertension* 24:1073-1079.

27. Netea RT, Smits P, Lenders JW, Thien T (1998): Does it matter whether blood pressure measurements are taken with subjects sitting or supine? *Journal of hypertension* 16:263-268.

28. Eşer İ, Khorshid L, Yapucu Güneş Ü, Demir Y (2007): The effect of different body positions on blood pressure. *Journal of clinical nursing* 16:137-140.

29. Netea R, Lenders J, Smits P, Thien T (2003): Both body and arm position significantly influence blood pressure measurement. *Journal of human hypertension* 17:459.

30. Sala C, Santin E, Rescaldani M, Cuspidi C, Magrini F (2005): What is the accuracy of clinic blood pressure measurement? *American journal of hypertension* 18:244-248.

Debriefing Transcriptions for the Question:

What was it like to experience internal body sensations like your breath and heartbeat?

The heartbeat was kinda interesting. At first, it kinda was weird. You know, and ‘cause when I was little, we lived in a two-story home, and when I would lay there in bed, my heart would scare me. I thought it was someone coming up the stairs. So I’ve never been a big fan of a heartbeat. But I kinda enjoyed it. Once I got the rhythm of the breathing and the heart and all that kinda stuff. I think I could hear it more being in the pool. And so that just kinda made it easier for me to concentrate on my breathing ‘cause I could really hear it.
—*Subject 1*

It made it a lot easier to focus on them much better. It didn’t bother me. I actually—even though I replied, ‘No’ to your survey, I just didn’t want to have to go through and explain all the times that I’ve tried to do the stupid meditation. Meditation is no fun. It’s not easy. And I don’t wanna do it! [laughs] But that was much easier! It was much easier, ‘cause you’re perfectly relaxed. You’re not sitting there going, ‘Do I gotta sit? Do I gotta lay down?’ Like when you try to meditate, a lot of times, it’s kinda like, ‘Oh my…’ I mean I can’t, even for a minute, meditate. Well, and all that is, truthfully, is trying to focus—still your mind and focus on your breath and that kind of thing. Way easier in there!
—*Subject 2*

I felt like I was just so disconnected. They never even crossed my mind. It was like everything just kinda disappeared. There wasn’t really any feeling of anything.
—*Subject 3*

Helped things slow down even further, going in breathing in the nose, out the mouth. Good. I mean it always feels good to feel it without pain.
—*Subject 4*

It was great! It was. I carry a lot of tension in my hips, so I was able to breathe and then hear my breath, and actually feel my breath going all the way into those areas. You know, if you just focus on where the tension is or where the pain is, and you just send your breath to that area. And just hearing it like that, it made the experience much more real. Calming. It’s calming. It’s just a grounding feeling.
—*Subject 5*

It just seemed like it was more intense. Like the breathing was louder and the heartbeat was louder.
—*Subject 6*

It wasn’t anything spectacular. When I have panic attacks, I can hear my heartbeat really loud so. I mean I could hear it. It wasn’t beating as fast as it would if I was having a panic attack. I could just like hear it slowing down and I could control it kind of, sort of.
—*Subject 7*

A little weird. I really don’t like it! It’s just really intense. You can hear it a lot louder than after running upstairs. That’s why I say it helps if you have a little music [laughs].
—*Subject 8*

It's calming. Like it's just kinda refreshing 'cause you're not thinking about anything else but the sound. Like that's usually how I calm myself down anyways is to listen 'cause my mom always said, 'Just breathe.' So that's what I do. [laughs]
—*Subject 9*

Well I was thinking about that beforehand, and you know, I was thinking, you know, my body was pretty noisy. Like I know I'm hungry, my stomach was making growling noise and then, yeah, it's hard for me to kind of notice my heartbeat. You know, I've been asked to do that before and it's kind of like challenging for me, I guess. But I mean I was able to hear it, you know, I don't know, in my ears, and it was almost like a—I don't know, just—and then, yeah, breathing for sure. It didn't bother me at all. I think hearing my heart 'cause it would go through my head, you know, kinda irritated me 'cause it was like a throbbing. Like a, 'Boom! Boom!'
—*Subject 10*

It was like weird at first, but after a little bit, you kinda get used to it. It was kinda comforting almost.
—*Subject 11*

It was really weird. [laughs] They were like—I could really—I was acutely aware of my breath. And with my breath, came, you know, a heightened awareness of my heartbeat. So, I kind of was just kind of trying to focus on that quite a bit to kind of like silence the chatter. But it was really kinda neat; kinda weird. I could really hear and feel my breathing sensations.
—*Subject 12*

Yeah, I kept thinking, 'I wonder if he can hear me breathing. I'm breathing really loud.' [laughs] I can't make my breath stop being so loud. Too much breathing. [laughs]
—*Subject 13*

That was weird! That was. Because I've done other things and tried to hear it, you know? I could really hear it. I mean, really, I could. I could really hear my breathing. I could hear it, and I could hear my heartbeat and I was like, 'Wow, that is weird.' It's weird 'cause I wasn't trying to. It just did. It was relaxing. At first, it was kinda scary. I think just because so much stress is on me today with the day I was having. At first, I thought I was having a panic attack. But then, I just kinda took over and relaxed and said, you know, 'Just relax. Don't think of anything and just let it.' And it was really neat! I could actually hear my heartbeat and stuff going on in my—it was weird!
—*Subject 14*

Yeah, even on the survey, I didn't really know how to explain it because it—I really felt and was one with my heartbeat and I could hear it, but it wasn't like the anxiety kind of experience as when I'm having an anxiety or panic attack. So it wasn't a negative thing. It was very in tune with myself kind of feeling. It's a lot different than anxiety and depression. It's more just getting to know yourself; being more in tune. And it's a positive, it is!
—*Subject 15*

I did also test like breathing through my mouth mostly, or my nose. My nose is kinda stopped up, so I mostly breathed through my mouth. But yeah, I think not having like a lot to look at or hear, my focus was more concentrated on my breathing than normally. I was able to like gauge, you know, how much I was breathing, or how long more so than I would just in a normal day. I kind of liked it. It was nice! I just

felt like more in tune with my body, like more aware of what was going on so that I could react immediately. Like, ‘Oh, I’m maybe hungry’ or like if my shoulder popped, I could sense where it was and everything. So I enjoyed being a little more aware.
—*Subject 16*

It was definitely more intense, especially when I focused in on my breathing. My heartbeat, I felt that pretty intensely too. So normally, going throughout the day, I don’t pay attention to it. I don’t notice it. But while being in there, it was very noticeable. My heartbeat was very, very noticeable. I usually don’t ever feel my heartbeat during the day. And yeah, I really felt it. And it almost felt like I could hear it also. So it’s pretty intense.
—*Subject 17*

It was kinda weird, but it’s kinda neat. It was interesting.
—*Subject 18*

It was interesting! You know, hearing my own—you know, I was thinking about, ‘Okay, how do my breath sounds sound? Is there anything unusual about them? So I found myself getting distracted by just listening to my own heartbeat and breathing. It was interesting to hear them and not see that my mind just kinda tosses them aside as being essentially, you know, ‘Okay, yeah, this is supposed to be there. We need to focus on something else.’ That is gonna, you know, be more engaging. So I noticed that, even though I could hear myself breathing. I could hear my, you know, internal pulse, but my heartbeat kinda fell into the background. You know, I could hear it fine if my mind really wanted to find it. But what I found was it pretty much defaulted to being pretty much focused on my respiratory pattern.
—*Subject 19*

It was intense. The breathing was pretty intense. And I was very aware of when I would take that sigh breath. And that was—I mean I know that we do it, but I’m usually not conscious of it. And then my stomach would—or my throat would make little gurgle sounds and I don’t really ever hear that. It was interesting ‘cause I normally don’t pay attention to my breathing. But in the water, you can hear. It’s really magnified. But it made me concentrate more on it.
—*Subject 20*

That was pretty cool. And I felt like there was a lot less tension involved with that. It was really weird. There was a point at which I felt like my heartbeat is my whole body moving in the water, like to the rhythm. It’s like waves or something. It was pretty cool. It was almost like all over. That feels pretty good!
—*Subject 21*

So my heart was one that was kind of giving me a slight bit of anxiety because, you know, being that still, you can feel everything, and it felt like I may not breathe enough to keep it at a steady rate. [laughs] I was worried about my heart rate going too low. That was really the only thing. I do notice how slow my breathing is! I kind of—I realize, you know, I don’t breathe that much when I’m relaxed. I feel like I have to like pay attention to it.
—*Subject 22*

It was very nice! I know a lot of the questions asked about digestive, and I don’t think—I apparently am not in tune with my digestive tract. I just don’t think about it! But it was neat. I mean being able to hear your heartbeat in your ears. But lots of breath-focusing, counting every progressive muscle relaxation technique that I could think of; kinda went through them all. And being able to do it without really any guilt to go through it ‘cause

there was nothing else I was supposed to be doing.
—*Subject 23*

I think because I'm already probably more aware of it, maybe, then it doesn't distract me or doesn't shock me or it doesn't seem weird or strange. Having the level of anxiety that I have, sometimes, I don't have to be floating in a tank of quietness [laughs] to feel my heartbeat; but it wasn't a panic feeling. It wasn't like noticing that my heart was racing kind of a panic. It was just there, just aware of it. So it wasn't distracting or anything.
—*Subject 24*

The breathing was crazy ‘cause it felt so deep. Like I felt like it wasn't—it was like past my body. Like it was going down and around me. And I felt—like I couldn't tell what part of my body was in the water and what part of my body wasn't in the water. I definitely could feel my whole body take it in and out. And like the heartbeat, because of the blood pressure, that made me realize I was calmer. ‘Cause I had a moment, there of stressful craziness, but [laughs] I was like, ‘You need to calm yourself down. Just feel it. Feel it through.' So I feel like I felt it more, definitely. Like my heartbeat and breathing.
—*Subject 25*

Well, you know, really the only thing that I even noticed was my breath. I didn't notice anything else. But then, at some point, I was—I think it might be because of allergies and lying down with my head back—it became difficult to breathe through my nose. So I started through my mouth at some point.
—*Subject 26*

Normally, I'm pretty good with as far as—I don't do it a lot, but I know about diaphragmatic breathing, and I've done it before, so I am familiar with that aspect. I don't practice it near as much as I should. As far as heartbeat, that was different! ‘Cause I don't really ever—it doesn't really seem like—or feel like my heart or anything—whatever, you know, day-to-day stuff, you know? Unless you've just got done working out or something like that! Whenever I'm out at the pool. Like right now, I couldn't tell you what my heartbeat is at all; but in the pool, it was kinda neat to actually, you know, feel it! Because you can be aware of it! But the breath is good because that's, of course, you have your breathing techniques and diaphragmatic breathing and stuff like that. So that kind of stuff works for me a little better normally. For some reason, like diaphragmatic breathing and some breathing exercises will give me a little bit more anxiety. But this time, not so much. So whenever I'd focus on breathing or whatever—so it was nice.
—*Subject 27*

Yeah! What's interesting is like I didn't have a lot of like—other than my breath; I noticed that I could feel and hear my breath a lot more if I breathed through my nose. And so I was like, ‘Well, I'll just breathe through my mouth so that I don't have to hear it.' Or feel it or whatever! And then, I started to kind of just realize that—I don't know—like I started breathing through my nose—just my nose—and even though I could hear it, it was somehow soothing. And then, that's what led to me being very still and then going into that—I don't know—whatever state it was, yeah. And for whatever reason that—I don't know if that played a role in today, but a couple times, I woke up and my throat was really relaxed—almost too relaxed. And so I had to like make myself breathe deep breaths. It was like I wasn't breathing. It was like I was kind of—I was breathing, but I wasn't breathing strongly, or with purpose. It was

more like—it's hard to explain—it was starting to like almost shut off. So I had to like make myself take a deep breath and then make myself breathe. It happened a couple times.
—*Subject 28*

I wasn't really focused on anything. My mind was completely blank. I didn't really hear my heartbeat too much, but I could feel and hear my breath a lot better. I think that's kind of why I feel so relaxed, 'cause my breathing was a lot more regulated like it should be.
—*Subject 29*

It was kinda neat because I'm so busy and my mind is going so many different places, I don't really notice it as much during the day. And I think I usually think that I have very much social awareness and awareness of my body because I'm so self-conscious a lot of times. But I've found that in the past, there's a lot of times I'm not totally aware. And I will have those reactions that I think I've got it under control [laughs] and really everybody sees it but me. And in there, just being so much more body-aware is just very different for me. The only time I usually notice my heart as much, is when I have very high—you know, my anxiety is pretty high or if I've just walked a flight of stairs, you know? [laughs] It's either the physical or that, you know, anxiety—matter of fact, I've been at the hospital a few times in the past, probably 15 years or so, thinking I was having a heart attack when it was really anxiety. And the weirdest part is my body will mimic these reactions. Like my fingers will tingle, and I think my hand's going numb, and you know, all these just manifestations of this anxiety. It's crazy. You know, I would notice my body some. I could feel my body, but I would be so relaxed, it was almost like, at times, my body was one with the water.
—*Subject 30*
